# A SPRY1 domain cardiac ryanodine receptor variant associated with short-coupled torsade de pointes

**DOI:** 10.1038/s41598-021-84373-9

**Published:** 2021-03-04

**Authors:** Zahia Touat-Hamici, Malorie Blancard, Ruifang Ma, Lianyun Lin, Yasmine Iddir, Isabelle Denjoy, Antoine Leenhardt, Zhiguang Yuchi, Pascale Guicheney

**Affiliations:** 1 INSERM, UMRS 1166, Faculté de Médecine Sorbonne-Université, Unité de Recherche sur les Maladies Cardiovasculaires et Métaboliques, 91, boulevard de l’Hôpital, 75013 Paris, France; 2 Institute of Cardiometabolism and Nutrition (ICAN), Sorbonne Université, Paris, France; 3 Department of Pharmacology, Northwestern University Feinberg School of Medicine, Chicago, IL USA; 4 Tianjin Key Laboratory for Modern Drug Delivery & High-Efficiency, Collaborative Innovation Center of Chemical Science and Engineering, School of Pharmaceutical Science and Technology, Tianjin University, Tianjin, 300072 China; 5 Département d’Oncologie Pédiatrique Laboratoire RTOP «Recherche Translationnelle en Oncologie Pédiatrique»-INSERM U830, Institut Curie, Paris, France; 6 Département de Cardiologie et Centre de Référence des Maladies Cardiaques Héréditaires, AP-HP, Hôpital Bichat, 75018 Paris, France; 7 Université de Paris, INSERM, U1166, 75013 Paris, France

**Keywords:** Cell biology, Genetics, Cardiology, Diseases

## Abstract

Idiopathic ventricular fibrillation (IVF) causes sudden death in young adult patients without structural or ischemic heart disease. Most IVF cases are sporadic and some patients present with short-coupled torsade de pointes, the genetics of which are poorly understood. A man who had a first syncope at the age of 35 presented with frequent short-coupled premature ventricular beats with bursts of polymorphic ventricular tachycardia and then died suddenly. By exome sequencing, we identified three rare variants: p.I784F in the SPRY1 of the ryanodine receptor 2 (RyR2), p.A96S in connexin 40 (Cx40), reported to affect electrical coupling and cardiac conduction, and a nonsense p.R244X in the cardiac-specific troponin I-interacting kinase (TNNI3K). We assessed intracellular Ca^2+^ handling in WT and mutant human *RYR2* transfected HEK293 cells by fluorescent microscopy and an enhanced store overload-induced Ca^2+^ release in response to cytosolic Ca^2+^ was observed in RyR2-I784F cells. In addition, crystal structures and thermal melting temperatures revealed a conformational change in the I784F-SPRY1 domain compared to the WT-domain. The novel RyR2-I784F variant in SPRY1 domain causes a leaky channel under non-stress conditions. The presence of several variants affecting Ca^2+^ handling and cardiac conduction suggests a possible oligogenic origin for the ectopies originating from Purkinje fibres.

## Introduction

Idiopathic ventricular fibrillation (IVF) is a leading cause of unexplained sudden cardiac death (SCD) in the absence of structural heart disease, particularly in young adults^1^. Current guidelines define IVF as a diagnosis of exclusion in patients who have survived a VF episode without any identifiable structural or metabolic cause, as assessed by clinical evaluation of known cardiac, respiratory, metabolic and toxicological etiologies that may lead to cardiac arrest^2^. It occurs in 5–10% of patients resuscitated from out‐of‐hospital cardiac arrest without clinical evidence of heart disease^3,4^. Most IVF patients are sporadic cases but a subset of patients have a family history of sudden cardiac death, suggestive of genetic origin^5^. Genetic screening of known genes responsible for arrhythmias led to the identification of only a few variants in a small percentage of cases^6^. This suggests that these patients are genetically heterogeneous and also that the IVF is likely oligogenic in origin, which could explain the low penetrance in the families. Multiple genetic variants may be causal, as is the case for other channelopathies^7^.

We focused our study on a patient with short-coupled torsade de pointes (scTdP), a ventricular tachycardia with a characteristic rotation of electrical activity around the isoelectric line. Contractions are non-uniform but organized, with a change in amplitude over time, where the first beat is characterized by a very short coupled interval (280–300 ms)^2^. It evolves, in most cases, to VF causing SCD. Most scTdP originate from Purkinje fibres^1,4,6,8,9^. In the first reported series of 14 patients, 30% of scTdP patients had a family history of sudden death, suggesting a genetic susceptibility to this pathology^10^.

The origin of the scTdP is currently unknown. Nevertheless, verapamil, a Ca^2+^-blocking agent, reduces arrhythmias in this condition but remains insufficient to eliminate the risk of sudden death^10,11^. This suggests that, at least in some patients, ectopies may occur in a context of Ca^2+^ deregulation. Interestingly, several variants have been identified recently in the ryanodine receptor 2 (RyR2) gene in scTdP patients^12–14^.

The ryanodine receptor 2, RyR2, is a large tetrameric Ca^2+^ release channel located in the cardiomyocyte sarcoplasmic reticulum that governs the Ca^2+^ release involved in the excitation–contraction coupling (ECC)^15^. Its gating properties are modulated by numerous accessory proteins, such as the 12.6-kDa FK506-binding protein (FKBP12.6)^16–18^. RyR2 mutations can result in severe disease phenotypes, and more than 150 mutations caused catecholaminergic polymorphic ventricular tachycardia (CPVT), and a few have been linked to arrhythmogenic right ventricular cardiomyopathy type 2 and idiopathic ventricular fibrillation^19^. The previously reported mutations are dominant missense mutations mostly located in specific regions or hotspots in the N-terminal, central, and C-terminal domains including the channel region^20^. HEK293 cells have been the primary cell type model used to study *RYR2* variants^21,22^. In cardiomyocytes, we can observe the ECC and also a mechanism called ‘store-overload induced Ca^2+^ release’ (SOICR), described by the team of SR Wayne Chen as the spontaneous release of Ca^2+^ through RyR2 which occurs when the sarcoplasmic reticulum store Ca^2+^ content reaches a critical level^23^. These events can be observed as sparks in cardiomyocytes and show up as oscillations in the whole cell in HEK293 cells. Sparks can trigger Ca^2+^ waves which will activate the Na^+^–Ca^2+^ exchanger. This activation will lead to an inward Na^+^ current which induces membrane depolarization and creates delayed after depolarization (DAD). If the amplitude of these DADs is sufficiently high, it may trigger a new action potential and then arrhythmias. It was reported that HEK293 cells expressing RyR2 displayed SOICR in a manner virtually identical to that observed in cardiac cells^22^.

In the heart, gap junctions (connexins or Cx) are intercellular channels that mediate exchange of ions and small molecules and mediate direct electrical coupling between cardiomyocytes, allowing rapid propagation of action potentials in the atria and ventricles^24^. In human cardiomyocytes, the principal connexins are Cx40, Cx43, and Cx45, expressed at different relative levels in a chamber-related manner^25^. Cx40, also called gap junction protein α 5 (GJA5), is not only the main connexin isoform in atria followed by Cx43, but is also highly expressed in Purkinje fibres^26,27^. Because the normal heart rhythm depends in part on the coupling of cardiomyocytes by connexins, any alteration in connexin functions may contribute to arrhythmias^25^.

In the present study, we showed for the first time that a RyR2 variant localized in the SPRY1 domain affects the channel structure and function, and that, in association with gap junction alteration in Purkinje fibres, it may be responsible for the patient’s tachycardia and ventricular fibrillation.

## Results

### Clinical phenotype

**Figure 1:**
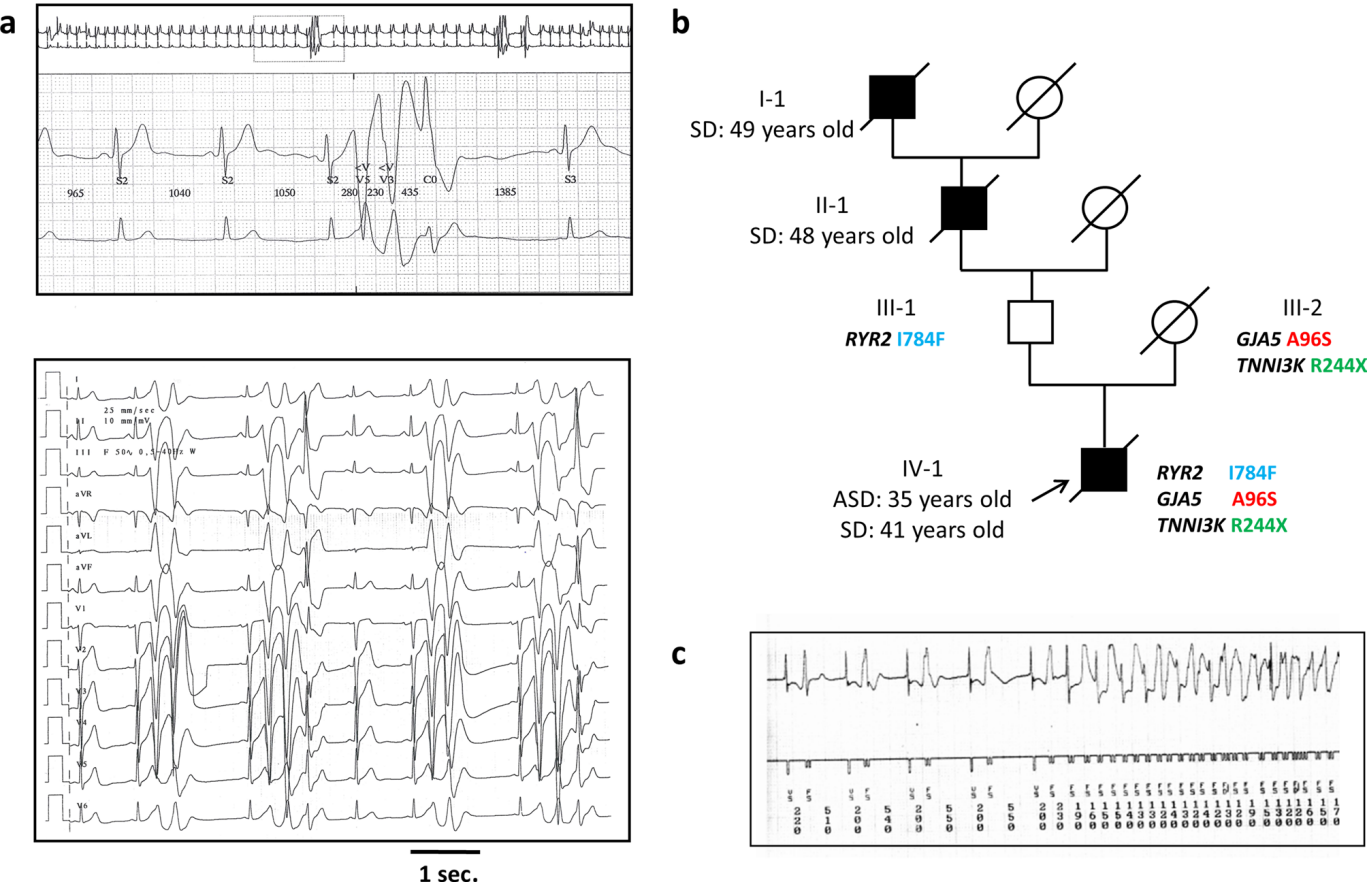
Pedigree and segregation of three variants. (**a**) Proband ECGs showing couplets and triplets of polymorphic ventricular beats. The coupling interval of the first ventricular premature beat is consistently very short: 280 ms. (**b**) Family pedigree and genotypes. All subjects were heterozygous carriers of the variants. Black symbols indicate individuals affected by sudden death, with the proband marked with an arrow, square: man, circle: woman, oblique bar: deceased, grey symbol: potentially affected individual, white symbol: clinically unaffected individual. SD: sudden death, ASD: aborted SD. (**c**) Polymorphic ventricular tachycardia and ventricular fibrillation recorded by ICD device the day of the proband death.

The proband of Spanish origin had frequent palpitations, predominantly at rest, and a first syncope at the age of 35 years. He had a structurally normal heart and no coronary artery disease. His ECG showed sinus rhythm with normal QTc interval, and short-coupled premature ventricular beats (scPVB) with salvoes of polymorphic ventricular tachycardia (Fig. 1a). Noteworthy, the patient’s exercise-stress test was negative. The patient (VI-1) was treated with verapamil, a Ca^2+^ channel blocker (120 mg 3× daily then 240 mg 2× daily) and received an implantable cardioverter defibrillator (ICD) (Fig. 1b). He died suddenly at rest after lunch and in the absence of emotional stress at the age of 41 years, due to a ventricular fibrillation that could not be stopped because the battery of his ICD was depleted (Fig. 1c). His great-grandfather, I-1, experienced a sudden death at the age of 49. His grandfather, II-1, has a first cardiac arrest at age 48 and died suddenly three days later. His father and mother, III-1 and III-2, never had syncope. His father was treated with pindolol, a beta-blocker, starting at age 40 to prevent hypertension. His mother, who died at the age of 80, developed persistent atrial fibrillation. Her last and only available medical examination report at age 76 mentioned a dilated left atrium at echocardiography and absence of ventricular abnormalities (Fig. 1b).

### Genetic analysis: identification of rare variants

After whole exome sequencing of proband and his parents’ DNA, we searched for potential pathogenic rare variants with a minor allele frequency (MAF) less than 0.1% in genes linked to inherited arrhythmias and cardiomyopathies, and in additional candidate genes overexpressed in Purkinje fibres (see Supplementary Table S1 online). We identified three heterozygous variants, two missense in *RYR2* and *GJA5* and a nonsense in *TNNI3K*, which were confirmed by Sanger sequencing (see Supplementary Fig. S1 online).

**Figure 2:**
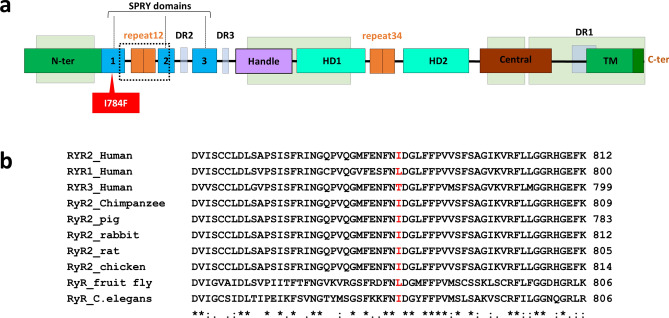
Localisation and evolutionary conservation of Ile784 in the RyR2 SPRY1 domain. (**a**) Schematic representation of RYR2 domains^20,30^. Dashed square shows the FKBP12.6 binding site to repeat12, SPRY1 and SPRY2 domains. Light green squares represent CPVT mutation hotspots: The N-terminal domain comprises amino acids 66-466 (CPVT-I), the central domain comprises amino acids 2246-2534 (CPVT-II) and the channel region comprises amino acids 3778-4201 (CPVT-III) and 4497-4959 (CPVT-IV). The I784F variant is located outside of the four hotspots where most previously reported mutations have been identified. N-ter: N-terminal; HD: Helical domain; TM: Transmembrane; DR: Divergent Region. (**b**) Sequence alignment of RyR2 proteins from several species and human RyR1 and RyR3 isoforms. It is noteworthy that the isoleucin is conserved in *Caenorhabditis elegans* (I2HAA6_CAEEL, encoded by unc-68).

A substitution was identified, c.2350A>T, in exon 21 of *RYR2* (NM_001035.2) changing an isoleucine to a phenylalanine at position 784, p.Ile784Phe (I784F). This variant, rs794728729, was reported in the Genome Aggregation Database (GnomAD Exomes, version 2.1.1) in two heterozygous subjects, leading to a minor allele frequency (MAF) of 0.0008% in the general population, but not found in GnomAD Genomes. The I784F variant is located in the cytosolic portion of RyR2 within the SPRY1 domain (Fig. 2a), which contains part of the binding site for FKBP12.6, a regulator of the channel opening probability^28^. The binding site of FKBP12.6 is formed by the handle, SPRY1 and SPRY3 domains. Of note, the I784F variant is not located directly in the FKBP12.6 binding site and is outside of the four hotspots (CPVT-I, II, III and IV) where most of the previously reported CPVT mutations have been identified (Fig. 2a)^20,28^. The isoleucine at position 784 is conserved through evolution in all mammalian RyR2s, but not in the other isoforms of RyRs, such as RyR1 and RyR3 (Fig. 2b). The high conservation of the region surrounding the isoleucine 784 suggests that this region could play an important functional role.

A second variant, c.286G>T (NM_181703.3) in exon 2 of *GJA5,* was identified leading to the p.Ala96Ser substitution (A96S, rs121434557). This variant has already been reported as pathogenic in a patient presenting with atrial fibrillation^29^. An equivalent variant chr1:147231060 GC ⇒ CT (Ala96Ser) is classified pathogenic by UniProt and confirmed using the American College of Medical Genetics (ACMG) classification. The MAF of rs121434557 is 0.0119% in GnomAD Exomes 2.1.1 and 0.0159% in GnomAD Genomes 2.1.

A third variant, c.781C>T (NM_015978.2) in exon 8 of *TNNI3K,* induced the formation of a stop codon at position 244, p.Arg244X (R244X). The MAF of this variant, rs56257331, is 0.0028% in GnomAD Exomes 2.1.1 and 0.00319% in GnomAD Genomes 2.1.

Sequencing his parents’ DNA showed that the father harbored the RyR2-I784F variant while the mother carried the two other variants, GJA5-A96S and TNNI3K*-*R244X (see Supplementary Fig. S1 online).

### RyR2-I784F variant exhibits an increased percentage of oscillating cells

**Figure 3:**
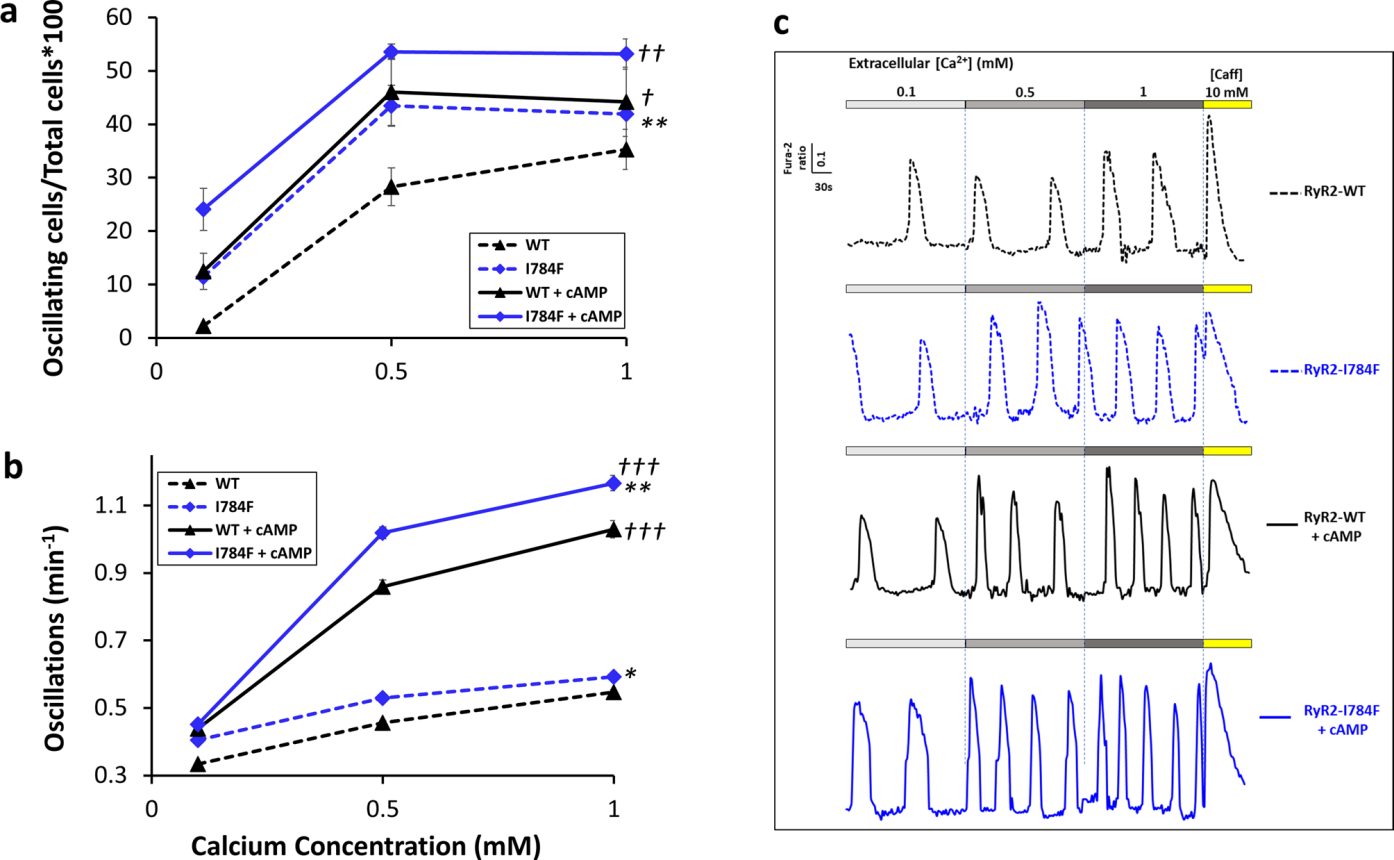
Enhanced Ca^2+^ release of RyR2-I784F mutant. HEK293 cells were transfected with eGFP- hRYR2-WT (black symbols) or eGFP- hRYR2-I784F (blue symbols) plasmids for 48 h and loaded with 5 μM Fura-2 for 45 min. Cells were then perfused with KRH buffer at 0.1, 0.5 or 1.0 mM CaCl_2_ (dashed lines). Solid lines correspond to the cells treated with cAMP for 20 min prior to the monitoring. (**a**) Percentage of oscillating cells. (**b**) Number of spontaneous Ca^2+^ oscillations per minute. (**c**) Representative traces of Ca^2+^ release under basal conditions and after cAMP treatment. The total numbers of cells analyzed for Ca^2+^ oscillations from three separate experiments under basal conditions were n = 588 for WT cells, and n = 524 for I784F cells; and after cAMP treatment, n = 347 for WT and n = 316 for I784F cells. Data are mean ± SEM. *p ≤ 0.02, **p ≤ 0.001, for RyR2-I784F vs WT-RyR2; ^*†*^*p* ≤ 0.02, ^*††*^*p* ≤ 0.001, ^*†††*^*p* ≤ 0.0001, for cAMP treatment vs basal conditions.

We aimed to study whether the RyR2-I784F variant could change the response of the channel to Ca^2+^ stimulation. Spontaneous Ca^2+^ release was investigated in HEK293 cells transfected either with eGFP-WT-hRYR2 or eGFP-I784F-hRYR2 plasmids. To assess whether the I784F variant affected the RyR2 adrenergic response, transfected cells were treated with 250 µM 8-bromo-cAMP (cAMP) 20 min before Ca^2+^ imaging. We first evaluated the percentages of oscillating cells and analyzed the data according to two-way ANOVA, the Tukey's post-hoc methodology was then used to identify significant differences between tested conditions while controlling for multiple testing. In Fig. 3a, we showed a difference in percentages of oscillating cells as a function of Ca^2+^ concentrations and cAMP treatment (for both p < 0.0001). Indeed, the percentages of oscillating cells were increased in RyR2-I784F expressing cells compared to RyR2-WT cells (p = 0.0004). This was observed under basal conditions (0.1 mM: 11.4 ± 0.6% vs 2.25 ± 0.18%; 0.5 mM: 43 ± 3.82% vs 28 ± 3.45%, and 1 mM: 41.9 ± 0.06% vs 35 ± 3.77%, p = 0.0027), as also, in cAMP treated cells (0.1 mM: 24.06 ± 3.93% vs 12.44 ± 3.38%; 0.5 mM: 53.57 ± 1.44% vs 46.04 ± 6.29%; and 1 mM: 53.14 ± 2.8% vs 44.20 ± 6.48%) even if the trend was less significant (p = 0.052). Note that the percentages of oscillating cells increased under cAMP treatment, both in RyR2-WT (p = 0.017) and RyR2-I784F (p = 0.0006) expressing cells, whatever the Ca^2+^ concentrations.

Nevertheless, when compared to basal conditions, the response of RyR2-I784F cells to cAMP treatment was lower than that of the RyR2-WT cells. Indeed, at 0.1 mM Ca^2+^, the percentage of oscillating cells was increased up to 5.52-fold in WT cells vs 2.11-fold in I784F cells (p = 0.0067). A difference was also observed at 0.5 mM, with a 1.64-fold increase in WT cells vs 1.24-fold in I784F expressing cells (p = 0.0272). By contrast, no difference was found at 1 mM (1.26-fold for WT and I784F; p = 0.0743).

These results show that the expression of the I784F variant increases the percentage of oscillating cells under basal conditions at 0.1 mM and 0.5 mM Ca^2+^, compared to WT. However, cAMP treatment induces a milder increase in percentages of oscillating cells in I784F cells, especially at low Ca^2+^ concentrations. These data suggest that the presence of the I784F variant may alter the response of RyR2 to cAMP.

### RyR2-I784F variant exhibits a higher spontaneous Ca^2+^ oscillation rate

We then assessed the effect of RyR2-I784F variant on the store-overload induced Ca^2+^ release (SOICR) which can be observed as spontaneous Ca^2+^ oscillations in transfected HEK293 cells. Using a two-way ANOVA test, we found that Ca^2+^ oscillation frequencies differed as a function of expressed variant (RyR2-I784F vs RyR2-WT; p < 0.0001), Ca^2+^ concentrations (p < 0.0001) and cAMP stimulation (p < 0.0001) with strong relationship between Ca^2+^ concentration and cAMP treatment (Fig. 3b,c).

RyR2-I784F expressing cells exhibited an increase in oscillation frequencies compared to RyR2-WT cells under basal conditions (p = 0.0203) and after cAMP treatment (p = 0.0012), especially at higher Ca^2+^ concentrations. Indeed, while there was no significant difference at 0.1 mM under basal conditions (0.40 ± 0.001 for I784F vs 0.33 ± 0.004 for WT) and after cAMP treatment (0.45 ± 0.001 for I784F vs 0.43 ± 0.009 for WT) between the two types of cells, the RyR2-I784F cells displayed a significant increase in oscillation frequencies at 0.5 mM of Ca^2+^ both under basal conditions and after cAMP treatment (basal: 0.53 ± 0.007 vs 0.45 ± 0.007. cAMP: 1.02 ± 0.017 vs 0.86 ± 0.019; p < 0.0001). The oscillation frequencies of RyR2-I784F were also significantly enhanced at 1 mM of Ca^2+^ under basal conditions and after cAMP treatment (basal: 0.59 ± 0.007 vs 0.54 ± 0.007. cAMP: 1.16 ± 0.022 vs 1.02 ± 0.025; p < 0.0001). Moreover, oscillation frequencies increased after cAMP treatment, both in RyR2-WT and RyR2-I784F (both p < 0.0001) expressing cells, whatever the Ca^2+^ concentrations.

### RyR2-I784F effect on caffeine-induced Ca^2+^ release

**Figure 4:**
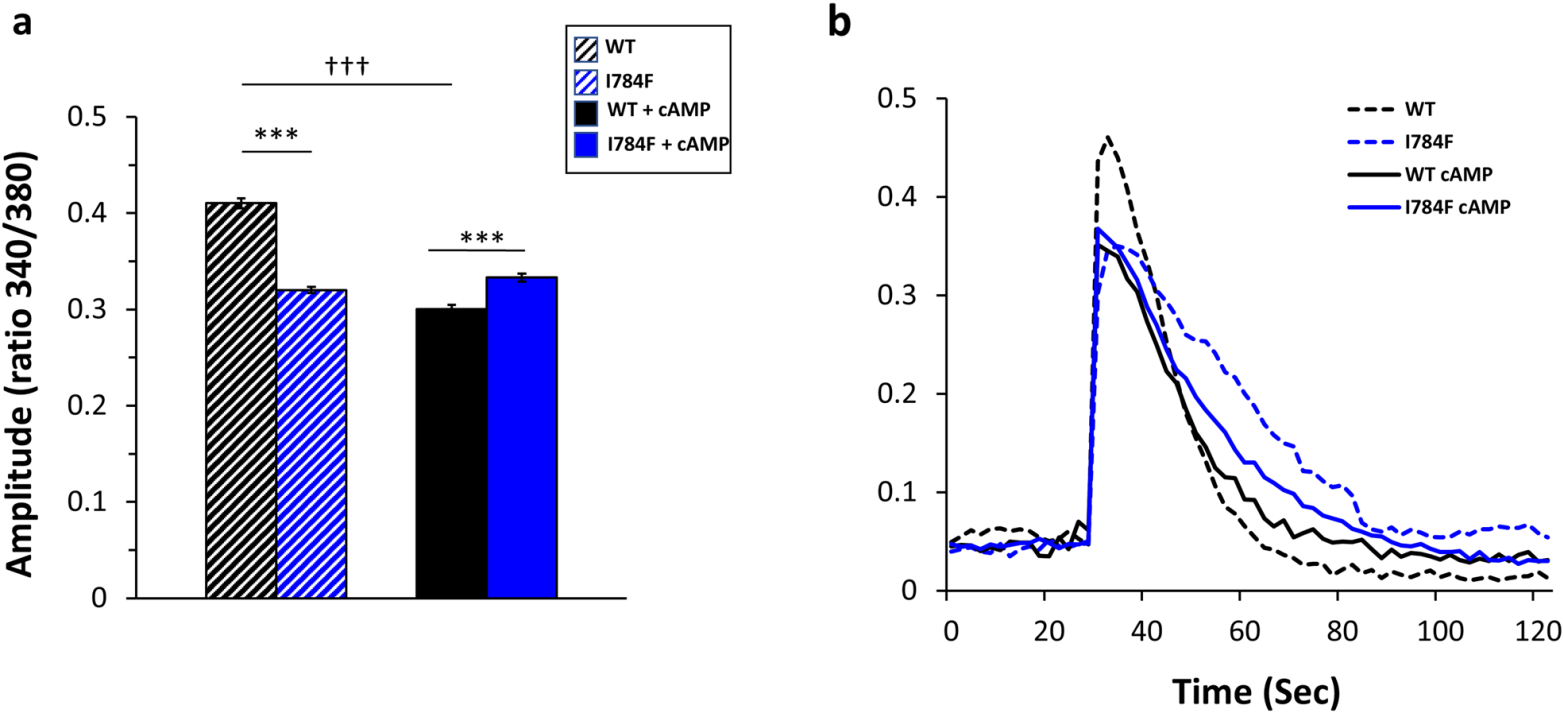
RyR2-I784F effect on caffeine-induced Ca^2+^ release. The Ca^2+^ store content was estimated by measuring the amplitude of caffeine (10 mM) induced Ca^2+^ release from oscillating cells in presence of 0.5 mM of Ca^2+^. (**a**) Graphical representation of the average from all cells. (**b**) Representative plots of one cell. The total number of cells from six separate experiments were for WT n = 1578 and for I784F n = 1760; and after cAMP for WT n = 957 and for I784F n = 1007. ^***^p < 0.0001 for WT-RyR2 vs RyR2-I784F; ^†††^*p* < 0.0001 for basal conditions vs after cAMP treatment.

Next, we aimed to determine whether the increase in oscillation frequencies impacts the total Ca^2+^ store. Total Ca^2+^ release was induced by the addition of 10 mM of caffeine, a known enhancer of the open probability of RyR2. The Ca^2+^ store content was estimated by measuring the amplitude of caffeine-induced Ca^2+^ release at 0.5 mM of Ca^2+^ (Fig. 4a,b). Ca^2+^ release in response to caffeine challenge was a function of the expressed variant (p < 0.0001) and cAMP treatment (p < 0.0001). Under basal conditions, the amplitude of caffeine-induced Ca^2+^ release was significantly reduced by about 20% in RyR2-I784F cells compared to RyR2-WT cells (0.32 ± 0.004 vs 0.41 ± 0.006, p < 0.0001).

After cAMP treatment, a significant reduction in caffeine-induced Ca^2+^ release was found in WT-RyR2 cells compared to basal conditions (0.30 ± 0.005 vs 0.41 ± 0.006, p < 0.0001). In contrast, no change was observed in RyR2-I784F cells (0.33 ± 0.001 vs 0.32 ± 0.004, p = 0.539, Fig. 4a,b), which could suggest again an altered response to cAMP (Fig. 3b). Under cAMP treatment, cells expressing RyR2-WT showed a slight, but significant, higher amplitude of caffeine-induced Ca^2+^ release (p < 0.0001).

### Crystal structure of RyR2 SPRY1 I784F

**Figure 5:**
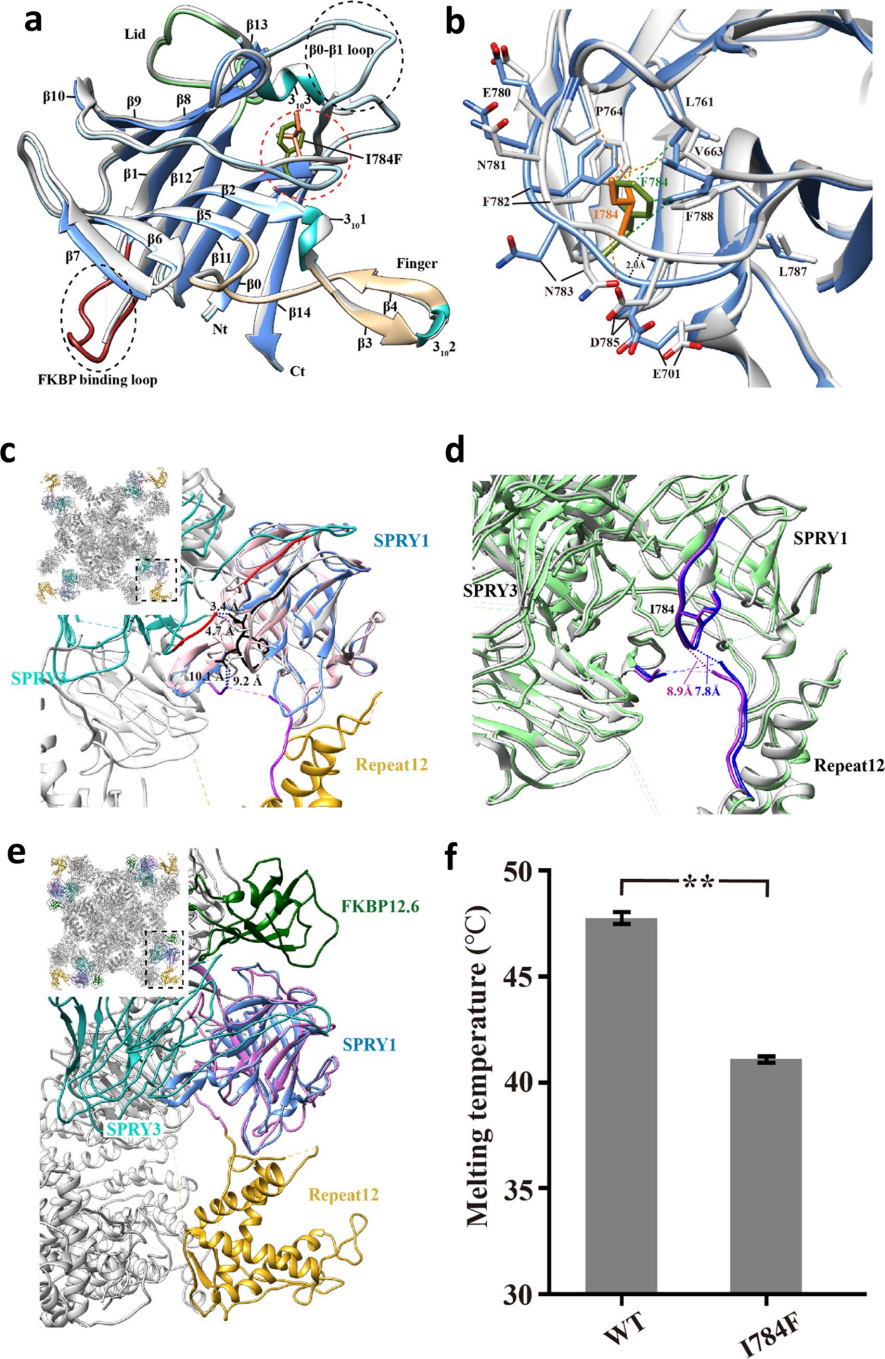
Crystal structures and thermal stability of RyR2-SPRY1. (**a**) Superimposition of the crystal structures of the WT RyR2-SPRY1 domain and I784F. WT is shown in white and I784F is colored according to different structural elements (β-strands in cornflower blue, 3_10_ helices in light sea green, the lid in light green, the finger in tan, the FKBP binding loop in orchid, and the other loops in light gray). The mutation site is shown as a stick. (**b**) Zoomed-in view of the loop containing I784F. WT is shown in white and I784F is shown in cornflower blue. (**c**) Docking of RyR2-SPRY1 WT (pink) and I784F (cornflower blue) structures to the cryo-EM structure of full-length pig RyR2 (PDB ID 5GO9) shows that loop 784 (black in I784F model) is in contact with a loop (red) from the SPRY3 domain (light sea blue) and close to a linker (purple) between the SPRY1 and Repeat12 (golden) domains. (**d**) Superimposition of the closed (PDB ID 5GO9) and open (PDB ID 5GOA) cryo-EM structures of full-length RyR2 shows that the SPRY1-Repeat12 linker moves closer to I784 upon gating. (**e**) Docking of RyR2-I784F-SPRY1 (cornflower blue) structure to the cryo-EM structure of full-length rabbit RyR1 (PDB ID 5TAQ) shows that loop 784 is not in direct contact with FKBP12.6 (green) but the other face of SPRY1 domain is. Figure was created using UCSF Chimera 1.14 (https://www.cgl.ucsf.edu/chimera). (**f**) Comparison of the melting temperatures of WT- and I784F-SPRY1. Error bars show the standard errors, with n = 4 for each group and **P < 0.0001 (two-tailed student *t*-test).

We solved the crystal structure of the RyR2 SPRY1 domain with the variant I784F at 1.45 Å resolution (see Supplementary Table S2 and Supplementary Fig. S3 online). There are two molecules in the asymmetric unit. We chose chain B for the structural analysis because the mutation site in chain A is closed to a crystal contact. The overall structure of I784F is similar to that of the wild type, consisting of two antiparallel β-sheets, a lid region, a β-hairpin finger and three 3_10_ helices. In addition, the two flexible loops with no visible electron density in the WT, including in the FKBP-interacting loop, have clear density in the mutant structure (Fig. 5a)^28^. While the root mean-squared deviation (RMSD) between the WT and I784F structures is only 0.34 Å over 177 Cα atoms, I784F clearly changes the conformation of the loop where the mutation is located. The mutation site faces a hydrophobic pocket formed by loop 663, loop 764 and loop 784. To fit the bulky side chain of Phe784, the main chain of loop 784 shifts away from the pocket by ~ 2.0 Å. This also changes the conformations of several neighboring residues, including V663, E701, E780, N781, F782, N783, D785, L787 and F788 (Fig. 5b). While the side chain of Ile784 interacts with Val663, Pro764, Phe782 and Asp783, Phe784 only interacts with Leu761 and Phe788 (dotted green line) (Fig. 5b). We docked WT and mutant SPRY1 crystal structures into RyR2 full-length cryo-electron microscopy (cryo-EM) structures (PDB: 5GO9 and 5GOA)^30^. The conformation of loop 784 was similar between the WT SPRY1 crystal structure and the cryo-EM model of full-length RyR2, but differed in I784F (Fig. 5c). I784F is close to one loop linking the SPRY1 and Repeat12 domains and another loop from the SPRY3 domain (Fig. 5c), suggesting that the conformational change caused by the mutation might impact these inter-domain interactions and in turn affect channel gating. In detail, the mutation moves loop 784 1.3 Å closer to SPRY3 and 0.9 Å further from the loop linking SPRY1 and Repeat12 domains (Fig. 5c). Comparison of the opened and closed structures of RyR2 shows that upon channel opening SPRY1-Repeat12 linker clearly moves ~ 1.1 Å closer towards loop 784 (Fig. 5d), which suggests that this interface is important in channel gating. The SPRY1 domain forms a major part of the binding site for FKBP12^28^. In summary, although I784F is not located in the FKBP12-binding interface, the conformational change caused by the mutation would move SPRY1 closer to SPRY3, which might change the overall FKBP-interaction interface on RyR2 and still affect its binding allosterically (Fig. 5e).

### Thermal stability of RyR2 SPRY1 I784F

We measured the stability of I784F with a thermal shift assay. Surprisingly, the variant reduces the melting temperature of the domain by 7° (~ 41 °C vs ~ 48 °C for WT) (Fig. 5f), suggesting a strong destabilizing effect exerted by the variant. Because I784F Tm is close to physiological temperature, a significant percentage (~ 6.3% according to the melting curve) of the mutant domain might be unfolded in vivo*,* thus changing its local structure.

## Discussion

The origin of ventricular fibrillation in young adults without structural cardiac abnormalities is poorly understood. Here, we identified pathogenic variants in three proteins contributing to cardiac excitability and conduction in a patient who repeatedly developed ventricular tachycardia and scTdP at rest. We showed that the new RyR2 variant in SPRY1 domain affects the channel structure and function and proposed that the cardiac electrical instability of this patient leading to lethal ventricular arrhythmias could be due to the combined effects of at least two of these variants.

To date, research into the origin of sudden cardiac death and idiopathic ventricular fibrillation has only identified a limited number of variants in genes encoding ion channels and channel-related proteins. More recently, the importance of gap-junction proteins was evidenced by identification of Cx43 variants in sudden infant death^7,31,32^. Where a family history of sudden death suggestive of heritable factors is reported, the penetrance is usually partial and low, suggesting an oligogenic inheritance of these types of ventricular arrhythmias^7^.

ScTdP was first reported in the 1980s, and in 1994 a series of fourteen patients was described with coupling intervals of 240–300 ms initiating TdP at rest; one-third of these patients had a family history of sudden death^10^. DPP6, a protein associated with the I(to) current, was first shown to contribute to IVF and extremely short-coupling interval PVCs by affecting the unique and highly expressed transient outward current in cardiac Purkinje fibres^6,33^. Several RyR2 variants were reported in IVF patients with or without scTdP^12–14,19,22,34^.

Following exome sequencing we identified three variants of interest: (1) a novel *RYR2* variant that results in the substitution of isoleucine for phenylalanine at position 784 (RyR2-I784F), located in the SPRY1 domain, outside of the CPVT-associated RyR2 mutational hotspot regions and affecting an evolutionary conserved residue; (2) a missense variant in *GJA5* leading to the substitution of alanine at position 96 for a serine (GJA5-A96S), previously reported as pathogenic^29^; and (3) a nonsense variant in *TNNI3K* which results in a stop codon at position 244 (TNNI3K-R244X).

RyR2 mutations have largely been associated with CPVT characterized by stress-induced arrhythmias, and associated with an enhanced Ca^2+^ release under adrenergic stimulation^22,35,36^. However, the role of RyR2 in the pathogenesis of scTdP, characterized by arrhythmias at rest, in the absence of adrenergic stimulation, is less known. The first identified variant, RyR2-H29D, was found in a case of scTdP at rest; a functional analysis by single channel assays demonstrated that RyR2-H29D caused a leaky channel under non-stress conditions^12,37^. More recently, patient-specific human-induced pluripotent stem cell (hiPSC)-derived cardiomyocytes (CMs) with RyR2-H29D confirmed an aberrant sarcoplasmic reticulum (SR) Ca^2+^ leak through RyR2 under physiological pacing, pro-arrhythmic electrical phenotypes, impaired contractile properties, and abnormal post-translational modifications compared to isogenic controls^37^. Three other RyR2 variants were identified in scTdP patients, two of which displayed a slight increase in sensitivity to cytosolic Ca^2+^ (RyR2-V1024I and RyR2-N1551S) and one of which resulted in an almost complete loss of function (RyR2-S4938F)^13^. Recently, a RyR2-M995V variant was reported in a scTdP patient without functional validation^14^.

The novel variant that we identified in the proband, RyR2-I784F, is the first described in the SPRY1 domain of RyR2. To determine the potential pathogenicity of this variant, we determined the thermal stability and the crystal structure of the RyR2 SPRY1 domain containing the I784F variant. The thermal melting experiment showed a strong destabilizing effect of the variant, suggestive of misfolding of SPRY1 domain. The structural analysis revealed that I784F causes a conformational change in a loop located at the interface with SPRY3 and Repeat12. This change might also affect other RyR2 inter-domain interactions and alter channel gating, but it is currently not possible to determine the consequences of this variant on the full RyR2 conformation. In particular, SPRY1 and the SPRY3 domains form part of the FKBP12.6 binding^25^, and even if I784 is not directly located at the FKBP binding interface, the conformational change could also alter FKPB12.6 affinity.

If this variant is the first analyzed in the RyR2 SPRY1 domain, a variant in the RyR1-SPRY1 domain was previously identified in a patient with multi-minicore disease and was reported to affect the interaction between SPRY1 and SPRY2^28,38^.

To assess whether the RyR2-I784F variant affects channel activity, we expressed human RyR2-WT or RyR2-I784F proteins in HEK293 cells. By monitoring spontaneous RyR2 activity using Ca^2+^ imaging, we observed a significant increase in Ca^2+^ oscillation frequency and an increase in the fraction of oscillating cells in presence of different Ca^2+^ concentration and cAMP treatment in cells transfected with RyR2-I784F compared to WT. In addition, evaluation of the stored Ca^2+^ by addition of caffeine shows a reduction in the Ca^2+^ pool. These data suggest a gain of function at rest. The underlying mechanism could be a reduction in the SOICR threshold by an increased luminal Ca^2+^ sensitivity or a leaky channel due to a conformational change inducing FKBP dissociation. Interestingly, in cAMP-treated cells, which mimics adrenergic conditions, the Ca^2+^ release was slightly attenuated in RyR2-I784F cells compared to RyR2-WT cells, which is consistent with the occurrence of VT at rest but not under adrenergic stimulation, as well as with the normal stress test of the patient.

The RyR2-I784F variant was inherited from the proband’s father who never developed syncope or VT prior to the age of 80. Of note, the father remained asymptomatic, and may have been protected by beta-blocker treatment initiated at the age of 40. The paternal grandfather and the paternal great-grandfather died suddenly before the age of 50 and they were probably carriers of the RyR2 variant, which strengthens the likelihood of its intrinsic pathogenicity.

We found that RyR2-I784F channels have a propensity to more spontaneous oscillations, which could be explained by different mechanisms. There are three main theories to explain RyR2 gain of function variants^39^. The first theory, developed by Andrew Marks’ team, suggested that the dissociation of the stabilizing FKBP12.6 from the RyR2 channel would induce Ca^2+^ leaks during diastole^40^. This theory could explain the effect of our variant since it is located in SPRY1 close to one of the binding sites of FKPB12.6^28^. Investigation of our variant using crystallography also showed a conformational change in the SPRY3 domain which is another domain involved in FKBP12.6 binding^28^. These conformational changes might induce a reduced interaction between FKBP12.6 and RyR2, which would lead to an unstable channel that is more prone to leakage. The second theory, developed by Wayne Chen’s team, suggested that the enhanced sensitivity of the channels could be explained by a reduction in the threshold to the luminal Ca^2+^ triggering the SOICR. A third theory focused on domain unzipping was developed by Yamamoto’s team^41,42^. The increase in spontaneous oscillations of the RyR2-I784F channel associated with the reduction in the Ca^2+^ store could be due to a reduction of the SOICR threshold. The conformational change induced by the variant might alter FKBP binding and/or interdomain stability and thus the channel gating properties, which would be consistent with the abnormal spontaneous activity of RyR2.

As a conclusion concerning this RyR2 variant, the conformational change could be the only mechanism responsible for the abnormal activity of RyR2, but we cannot exclude the existence of two additive mechanisms.

Cardiac conduction is mediated by gap junction channels that are formed by connexin (Cx) protein subunits. The mammalian heart shows regional differences both in the connexin expression profile and in the degree of electrical coupling^43,44^. The latter reflects functional requirements for conduction velocity which needs to be low in the sinoatrial and atrioventricular nodes and high in the ventricular conduction system. Cx40 and Cx43 play critical roles and their regional ratio is precisely regulated. Cx40 is expressed in atrial cardiomyocytes with Cx43, while Cx43 alone is expressed in working ventricular myocytes. Interestingly, Cx40 is highly expressed in the Purkinje network, with Cx45 at a lower level^25,43,45,46^ and absence of Cx43 in Purkinje fibres was showed by in situ hybridization^47^. These data were recently confirmed by single cell transcriptome performed by Goodyer and collaborators who identified Cx40 as the first specific marker of distal Purkinje fibres^27^. All these studies have been performed in different species, but studies on the human heart led to the same conclusion that Cx40 is Purkinje fibre-specific^26^. Numerous studies have shown that gap junction channel dysregulation could lead to arrhythmogenic disorders, including cardiac conduction abnormalities in mice lacking Cx40^44,45,48^.

The Cx40-A96S variant that we identified in the proband has already been characterized in a patient suffering from idiopathic atrial fibrillation, resulting in absent or weak electrical coupling^29^. Our patient inherited this variant from his mother, who developed late persistent atrial fibrillation and a dilated left atrium. Nevertheless, it is frequent that the same variant can give rise to different phenotypes depending of the genetic and physiological context. In the current study, we postulated that this variant could be a substrate for the development of ectopies from Purkinje fibres and scTdP in association with the RyR2-I784F variant. Of course, identification of other patients with such variants would be needed to strengthen this hypothesis.

TNNI3K is a cardiac-specific protein that plays a role in cardiac contractility^49^. Four mutations in this gene have been identified in patients with dilated cardiomyopathy and CCD^49,50^. TNNI3K-G526D was identified in a three-generation family of patients with CCD, atrial tachyarrhythmia and dilated cardiomyopathy vulnerability^49^. TNNI3K-T539A was identified in a young child with conduction abnormalities^51^. Then, a splicing variant, TNNI3K-c.333 + 2T>C, leading to haploinsufficiency was reported in patients with dilated cardiomyopathy and/or CCD and a history of syncope and sudden death^50^. More recently, a variant TNNI3K-E768K co-segregating in three large families, displayed enhanced kinase activity in contrast to the two preceding missense variants which were associated with reduced activity^52^. The pathophysiological consequences of these variants are probably not completely understood. Nevertheless, previous studies in mice demonstrated increased conduction indices and pro-cardiomyopathic effects with increased levels of Tnni3k while mice lacking Tnni3k did not show echocardiographic and electrocardiographic abnormalities, suggesting that it is not essential in this context, at least in mice^53,54^. Moreover, there are a relatively high number of *TNNI3K* nonsense variants in the populations according to ExAC database. In the present study, the proband’s mother was a carrier of the TNNI3K-R244X variant without conduction abnormalities. Altogether, these data suggest that TNNI3K nonsense variants might only be susceptibility factors for conduction abnormalities or arrhythmias but are not causal per se.

## Conclusion

Exome sequencing was performed in a patient presenting short-coupled ventricular tachycardia and sudden death at rest, consistent with the diagnosis of scTdP^6,10^. A growing body of evidence suggests that Purkinje fibres play a pivotal role in this rare condition^55,56^. A new RyR2 variant I784F in the SPRY1 domain was identified and shown to be pathogenic since it changes the thermal stability of the SPRY1 domain, its structure, and also increases the propensity to spontaneous Ca^2+^ release in human RyR2-I784F transfected HEK293 cells. Moreover, the patient was a carrier of a pathogenic variant in Cx40, a gap junction protein highly and specifically expressed in Purkinje fibres.

Arrhythmias are multi-factorial in origin, involving interplay between gap-junctional coupling, membrane excitability, and cell and tissue architecture. We hypothesized that the combined presence of variants that decrease the conduction velocity and increase sensitivity to Ca^2+^ could be responsible for cardiac instability, providing a reentrant path and anchoring reentrant waves, ultimately leading to VF and sudden death.

## Limitations

Our results have shown an enhanced spontaneous release of Ca^2+^ by the RyR2 I784F channel in HEK293 cells compatible with increased propensity for arrhythmias at rest. However, HEK293 cells lack many cardiac-specific proteins, and much remains to be done to fully understand the mechanism of this variant in cardiac cells. In particular, the absence of further enhancement of the Ca^2+^ release by adrenergic stimulation, as observed for CPVT RyR2 mutations, is an interesting topic to elucidate in order to gain an insight into the underlying mechanisms of scTdP RyR2 variants and this type of ventricular tachycardia originating from Purkinje fibres at rest. Mouse models harboring one or both RyR2 and Cx40 variants are presently unavailable and may not recapitulate the phenotype since extrapolation from mouse to man is limited because the mouse heart cannot accommodate large re-entrant circuits. Further comprehensive and detailed investigations will be required to address these important issues.

## Methods

### Study subjects

Clinical evaluation of the proband included 12-lead electrocardiogram (ECG), 24-h Holter ECG, exercise-stress test (EST), echocardiography, and coronary angiography. ScTdP was diagnosed according to previous descriptions^10^. Blood samples obtained after signed informed consent were collected for genetic analyses following the granting of the approval of the local ethics committee of Pitié-Salpêtrière Hospital. The study was conducted according to the principles of the Helsinki Declaration.

### Targeted exome sequencing

Library preparation, exome capture, sequencing and data analysis were performed by IntegraGen SA (Evry, France). Genomic DNA was captured using Agilent in-solution enrichment methodology (SureSelect XT Clinical Research Exome, Agilent) with their biotinylated oligonucleotides probes library (SureSelect XT Clinical Reasearch Exome—54 Mb, Agilent), followed by paired-end 75 base pair sequencing on an Illumina HiSeq4000 as previously described^17^. Base calling was performed using the Real-Time Analysis software sequence pipeline (2.7.6) with default parameters. Sequence reads were mapped to the human genome build (hg19/GRCh37) using Elandv2e (Illumina, CASAVA 1.8.2) allowing multiseed and gapped alignments.

### Construction of *RYR2* variants

The pcDNA3-eGFP-hRyR2 plasmid containing the human RYR2 sequence (X98330) was kindly provided by Spyros Zissimopoulos (UK). The five mismatches between the X98330 and the RyR2 reference sequences (NM001035.2, Q92736) were first corrected by oligonucleotide directed mutagenesis (QuikChange II Site-Directed mutagenesis Kit, Agilent Technologies): proline 1037 was changed to a leucine, and p.RTMRT at position 2785–2789 was replaced by p.WGWRI. To generate the I784F variant, a fragment of h*RYR2*, digested by SpeI and FspAI, was sub-cloned into pCR-Blunt, and the isoleucine 784 was replaced by a phenylalanine using mutagenesis. Then the mutated SpeI/FspAI fragment of the pCR-Blunt plasmid was amplified by overlapping PCR to be re-inserted into the full-length RYR2 plasmid using a recombination kit (NEBuilder HiFi DNA Assembly Cloning kit, New England Biolabs, France).

### Cell culture and transfection

Human embryonic kidney 293 (HEK293) cells were grown in Dulbecco’s Modified Eagle Medium (DMEM) (Life Technologies, France) supplemented with 10% FBS, 100 U/mL penicillin (1%), and 100 μg/mL streptomycin (1%). Cells were cultured in a humidified atmosphere at 37 °C, 5% CO_2_. Cell transfection was performed using Lipofectamine 3000 (Life Technologies, France) according to the manufacturer’s instructions. Briefly, one day before transfection, HEK293 cells were seeded in a 35 mm µ-dish with a glass bottom (Ibidi, CliniSciences, France), and grown to 60–70% confluency. HEK293 cells were transfected with 280 ng of eGFP-hRyR2-WT or eGFP-hRyR2-I784F plasmids.

To ensure that the RyR2-WT- and RyR2-I784F expression levels were similar, the protein levels of cell lysates were assessed by immunoblot forty-eight hours after transfection (see Supplementary Fig. S2a online) and the percentages of caffeine-responding cells with respect to total cells were determined (see Supplementary Fig. S2b online).

### Ca^2+^ imaging

Intracellular Ca^2+^ mobilisation in HEK293 cells was carried out 48 h after transfection using single cell Ca^2+^ imaging and the fluorescence Ca^2+^ indicator dye fura-2-AM as described previously^21^. Briefly, forty-five minutes prior to Ca^2+^ monitoring, cells were loaded with the fluorescent Ca^2+^ dye Fura2-AM (5 µM) (ThermoFisher Scientific, France) in Krebs–Ringer HEPES (KRH) buffer (in mM: 120 NaCl, 25 HEPES, 5.5 glucose, 4.8 KCl, 1.4 CaCl_2_, 1.2 KH_2_PO_4_, 1.2 MgCl_2_; pH 7.4) at 37 °C and 5% CO_2_. Cells were then perfused with KRH buffer containing different Ca^2+^ concentrations (0.1, 0.5 or 1 mM) at room temperature. At the end of each measurement, 10 mM caffeine was added to select only the responsive cells. Single-cell intracellular calcium measurements were performed in Fura-2-loaded cells using a Nikon Eclipse Ti-U inverted fluorescent microscope (Nikon, France) equipped with a dual excitation filter wheel (340 nm/380 nm), a 515-nm emission filter and a Hamamatsu ORCA-D2 CCD camera (Hamamatsu Photonics, France). Calcium traces were obtained by sequential acquisition of one image every 2 s for 3 min using a 20X S-Fluor 0.75 NA (WD = 1 mm) objective. Image acquisition was performed with the HCimage software (Hamamatsu, USA). Fluorescence intensities in single cells were determined using region of interest (ROI) analysis with ImageJ software (National Institutes of Health; https://imagej.nih.gov/ij/). Ca^2+^ signals were measured in the caffeine-responsive single cells using F340/380 ratios after subtraction of Fura-2 background fluorescence. For experiments with cAMP treatment, cells were pretreated with 250 µM of 8-bromo-cyclic AMP (Sigma-Aldrich-Merck, France) for 20 min. at room temperature before Ca^2+^-KRH perfusion.

### Caffeine-induced Ca^2+^ release in HEK293 cells

Transfected HEK293 cells were loaded with 5 µM of Fura2-AM for 45 min and then perfused with 0.5 mM Ca^2+^-containing KRH buffer. After 3 min, 10 mM caffeine was added in order to induce the total Ca^2+^ store release. The peak amplitude of each caffeine-induced Ca^2+^ release was determined in each cell using the F340/380 ratio. The total number of cells from six separate experiments were under basal conditions WT n = 1578 and I784F n = 1760, and after cAMP WT n = 957 and I784F n = 1007.

### Expression, purification and crystallization

The I784F mutation was introduced into the RyR2-SPRY1 domain (650–844) of a mouse construct within a pET28-HMT vector containing an N-terminal His-tag, a MBP-tag and a TEV cleavage site^57^. Protein was expressed and purified as previously described for the RyR2-WT-SPRY1^28^. Crystals were grown by hanging drop vapor diffusion at 4 °C. I784F-SPRY1 (10 mg/mL) was crystallized in 0.1 M sodium acetate (pH 4.5) plus 0.8 M sodium. Crystals were transferred to a solution supplemented with 30% isopropyl alcohol and flash-frozen in liquid N_2_ prior to data collection.

### Data collection and structure determination

Diffraction data was collected with the Shanghai Synchrotron Radiation Facility (SSRF) beamline BL18U1^58^ and processed using the HKL3000 data processing program^59^. The I784F structure was solved by molecular replacement in PHENIX using the wild-type mouse RyR2-SPRY1 domain (PDB ID 5C33) as the searching model^60^. Further model building was performed manually using COOT^61^. Structure refinement was performed using PHENIX with high-resolution (1.45 Å) native data. Data collection and final refinement statistics are available in Supplementary Table S2 online.

### Fluorescence-based thermal shift assays

The protein melting curves were measured using a fluorescence-based thermal shift assay^62^. Ten ml of protein (1 mg/mL) were diluted to 50 mL with a buffer containing 10 mM sodium phosphate (pH 7.4), 150 mM KCl, 14.3 mM beta-mercaptoethanol and SYPRO orange dye (Sigma-Aldrich, China). The melts were obtained using a QuantStudio 6 Flex real-time PCR machine (ThermoFisher Scientific, China), with the SYBR green filter option. The melting temperatures were held at 10 °C for 3 min., and then increased at a rate of 0.033 °C/s up to 95 °C. The melting temperatures were obtained by taking the midpoint of each transition.

### Statistical analysis

Data are expressed as the mean ± SEM. Differential analyses were performed using two-way Analysis of Variance (ANOVA) implemented in the R statistical environment. The Tukey's post-hoc methodology was then used to identify significant differences between tested conditions while controlling for multiple testing. Statistical analysis in Fig. 5 were performed using Student’s *t* test. A p value inferior to 0.05 was considered as significant.

## Supplementary Information

Supplementary Information.

## Data Availability

Atomic coordinates and structure factors for RyR2 SPRY1 I784F (PDB ID 6J6L) have been deposited in the RCSB Protein Data Bank.
