# Supplementary material for: A SPRY1 domain cardiac ryanodine receptor variant associated with short-coupled torsade de pointes
Source: Sci Rep. 2021 Mar 4;11:5243. doi: 10.1038/s41598-021-84373-9 (PMC7970841; doi:10.1038/s41598-021-84373-9)

**Supplementary data**

**A SPRY1 domain cardiac ryanodine receptor variant associated with short-coupled torsade de pointes**

Zahia Touat-Hamici^1,2^*, PhD, Malorie Blancard^1,2,3^*, PhD, Ruifang Ma^4^*, M.Sc, Lianyun Lin^4^, M.Sc, Yasmine Iddir^1,5^, Isabelle Denjoy^6,7^, MD, Antoine Leenhardt^6,7^, MD, Zhiguang Yuchi^4#^, PhD, Pascale Guicheney^1,2#^, PhD.

^1^ INSERM, U 1166, Unité de recherche sur les maladies cardiovasculaires et métaboliques, Paris, France;

^2^ Sorbonne University, UMRS 1166, Institute of Cardiometabolism and Nutrition (ICAN), Paris, France;

^3^ Department of Pharmacology, Northwestern University Feinberg School of Medicine, Chicago, IL, USA;

^4^ Tianjin Key Laboratory for Modern Drug Delivery & High-Efficiency, Collaborative Innovation Center of Chemical Science and Engineering, School of Pharmaceutical Science and Technology, Tianjin University, Tianjin, China ;

^5^ Institut Curie, Département d’Oncologie Pédiatrique Laboratoire RTOP « Recherche Translationnelle en Oncologie Pédiatrique » - INSERM U830, Paris, France ;

^6^ AP-HP, Hôpital Bichat, Département de Cardiologie et Centre de Référence des Maladies Cardiaques Héréditaires, F-75018 Paris, France ;

^7^ Université de Paris, INSERM, U1166, F-75013 Paris, France.

*Contributed equally

**Supplementary Table 1. Genes linked to inherited arrhythmias and cardiomyopathies, and additional candidate genes overexpressed in Purkinje fibers** (according to^1–6^). AF: Atrial Fibrillation, ARVC: Arrhythmic Right Ventricular cardiomyopathy, BrS: Brugada syndrome, CCD: Cardiac Conduction Disorder, CPVT: Catecholaminergic Polymorphic Ventricular Tachycardia, DCM: Dilated Cardiomyopathy, ERS: Early Repolarization Syndrome, HCM: Hypertrophic Cardiomyopathy, IVF: Idiopathic Ventricular Fibrillation, LQT: Long QT syndrome, LVNC: Left Ventricular Non-Compaction, SQT: Short QT Syndrome, SSS: Sinus Node syndrome.

| **Cardiac arrhythmias** | Pathologies | **Cardiomyopathies** | Pathologies | **Highly expressed in Purkinje fibers** |
| --- | --- | --- | --- | --- |
| ***ABCC9*** | BrS21, ERS, AF | ***ACTC1*** | HCM, DCM | ***ATP1A1*** |
| ***AKAP9*** | LQT11 | ***ACTN2*** | DCM, HCM, LVNC | ***ATP2B1*** |
| ***ANK2*** | LQT4 | ***ANKRD1*** | DCM | ***ATP2B4*** |
| ***CACNA1C*** | BrS3, ERS, LQT8, SQT4, IVF | ***BAG3*** | DCM | ***BMP2*** |
| ***CACNA2D1*** | BrS11, ERS, SQTS6 | ***CALR3*** | DCM | ***CACNA1D*** |
| ***CACNB2*** | BrS4, ERS, SQT5 | ***CDH2*** | ARVC | ***CACNA1G*** |
| ***CALM1*** | CPVT, LQTS, IVF | ***CRYAB*** | HCM | ***CACNA1H*** |
| ***CALM2*** | CPVT, LQTS | ***CSRP3*** | HCM | ***CACNA1I*** |
| ***CALM3*** | LQTS | ***CTNNA3*** | ARVC | ***CACNA2D2*** |
| ***CASQ2*** | CPVT | ***DES*** | HCM, DCM | ***CIC2*** |
| ***CAV3*** | LQT9 | ***DMD*** | DCM | ***CIC6*** |
| ***DPP6*** | IVF, ERS | ***DNAJC19*** | DCM | ***CPNE5*** |
| ***FGF12*** | BrS18 | ***DSC2*** | ARVC | ***ETV1*** |
| ***GPD1L*** | BrS2 | ***DSG2*** | ARVC | ***GJA1*** |
| ***HCN4*** | BrS8, SSS | ***DSP*** | ARVC | ***GJC1*** |
| ***GJA5*** | AF | ***EMD*** | DCM | ***HCN1*** |
| ***KCNA5*** | AF | ***EYA4*** | DCM | ***HCN3*** |
| ***KCND2*** | ERS | ***FHL1*** | HCM | ***HCN4*** |
| ***KCND3*** | BrS14, IVF | ***FHL2*** | DCM | ***ID2*** |
| ***KCNE1*** | LQT5 | ***FLNC*** | HCM | ***IGFBP5*** |
| ***KCNE1L*** | BrS13, IVF, AF | ***FKTN*** | DCM | ***IRX3*** |
| ***KCNE2*** | LQT6, AF | ***FOXD4*** | DCM | ***ITPR1*** |
| ***KCNN3*** | AF | ***JPH2*** | HCM | ***ITPR3*** |
| ***KCNE3*** | BrS6 | ***GLA*** | HCM | ***KCNA3*** |
| ***KCNH2*** | BrS9, LQT2, SQT1 | ***JUP*** | ARVC | ***KCNA4*** |
| ***KCNJ2*** | LQT7, SQT3, AF | ***LAMP2*** | HCM | ***KCNA6*** |
| ***KCNJ5*** | LQT13 | ***LDB3*** | DCM, LVNC | ***KCNC4*** |
| ***KCNJ8*** | BrS10, ERS | ***LMNA*** | DCM, ARVC | ***KCND1*** |
| ***KCNQ1*** | LQT1, SQT2, AF | ***MYBPC3*** | HCM | ***KCND3*** |
| ***MYL4*** | AF | ***MYH6*** | CMD, SSS | ***KCNE2*** |
| ***NPPA*** | AF | ***MYH7*** | HCM | ***KCNE4*** |
| ***NUP155*** | AF | ***MYL2*** | HCM | ***KCNJ3*** |
| ***PITX2*** | AF | ***MYL3*** | HCM | ***KCNK1*** |
| ***PKP2*** | BrS19 | ***MYLK2*** | HCM | ***KCNK3*** |
| ***RANGRF*** | BrS12 | ***MYOZ2*** | HCM | ***KCNK5*** |
| ***RRAD*** | BrS | ***MYPN*** | HCM | ***NKX2-5*** |
| ***RYR2*** | CPVT | ***NEXN*** | HCM | ***NTM*** |
| ***SCN10A*** | BrS22, ERS | ***PKP2*** | ARVC | ***PCP4*** |
| ***SCN1B*** | BrS5, AF | ***PLN*** | DCM, HCM, ARVC | ***RGS6*** |
| ***SCN2B*** | BrS17, AF | ***PRKAG2*** | HCM | ***RYR3*** |
| ***SCN3B*** | BrS7, AF | ***PSEN1*** | DCM | ***SCN2A*** |
| ***SCN4B*** | LQT10, AF | ***PTPN11*** | HCM | ***SCN9A*** |
| ***SCN5A*** | BrS1, LQT3, IVF, ERS, SSS, CPVT, AF | ***PSEN2*** | DCM | ***SEMA3*** |
| ***SLMAP*** | BrS15 | ***RBM20*** | DCM |  |
| ***SNTA1*** | LQT12 | ***SDHA*** | DCM |  |
| ***TECRL*** | CPVT | ***SGCD*** | DCM |  |
| ***TNNI3K*** | CCD | ***SYNE1*** | DCM |  |
| ***TRDN*** | CPVT | ***SYNE2*** | DCM |  |
| ***TRPM4*** | BrS20, CCD | ***TAZ*** | DCM |  |
| ***ZFHX3*** | AF | ***TCAP*** | DCM |  |
|  |  | ***TMEM43*** | ARVC |  |
|  |  | ***TMPO*** | DCM |  |
|  |  | ***TNNC1*** | DCM, HCM |  |
|  |  | ***TNNI3*** | DCM, HCM |  |
|  |  | ***TNNT2*** | DCM, HCM, LVNC |  |
|  |  | ***TP63*** | ARVC |  |
|  |  | ***TPM1*** | DCM, HCM, LVNC |  |
|  |  | ***TTN*** | HCM, DCM |  |
|  |  | ***TTR*** | HCM |  |
|  |  | ***VCL*** | HCM, DCM |  |

**Supplementary Table 2. Data collection and refinement statistics for the RyR2- I784F-SPRY1 crystal.**

| Crystal | RyR2- I784F- SPRY1 |
| --- | --- |
| λ for data collection (Å) | 0.9795 |
| **Data collection** |  |
| Space group | P2_1_2_1_2_1_ |
| *Cell deminsion (Å)* |  |
| a, b, c ( Å ) | 52.95, 64.38, 108.48 |
| α, β, γ, (°) | 90.00, 90.00, 90.00 |
| Resolution | 27.68 - 1.45 (1.50 - 1.45) |
| Rmerge† | 0.159 (0.702) |
| Average I/σ(I) | 12.44 (2.30) |
| Completeness (%) | 99.52 (96.43) |
| Redundancy | 6.0 (3.4) |
| Z | 2 |
| **Refinement** |  |
| Resolution | 27.68 - 1.452 |
| No. reflections | 65,901 |
| R_factor_/R_free_ (10% data) | 0.172/0.203 |
| RMSD length (Å) | 0.009 |
| RMSD angle (°) | 0.01 |
| **No. of atom** |  |
| Protein | 6482 |
| Ligands | 24 |
| Water | 508 |
| **Ramachandran plot (%)** |  |
| Most favored | 96.53 |
| Additionally allowed | 3.47 |

**Supplementary Figure S1. Sequence electropherograms of the proband and his family members**. **a)** DNA sequencing demonstrating a heterozygous single A to T nucleotide substitution at position 2350 of the *RYR2* gene leading to a I784F missense variant. The I784F mutation was also found in his father. **b)** DNA sequencing of *GJA5* gene demonstrating a heterozygous single G to T nucleotide substitution at position 286 leading to a A96S missense variant. The variant was also found in his mother. **c)** DNA sequencing of *TNNI3K* gene demonstrating a heterozygous single C to T nucleotide substitution at position 781 leading to an R244X nonsense variant. This variant was in his mother.

**
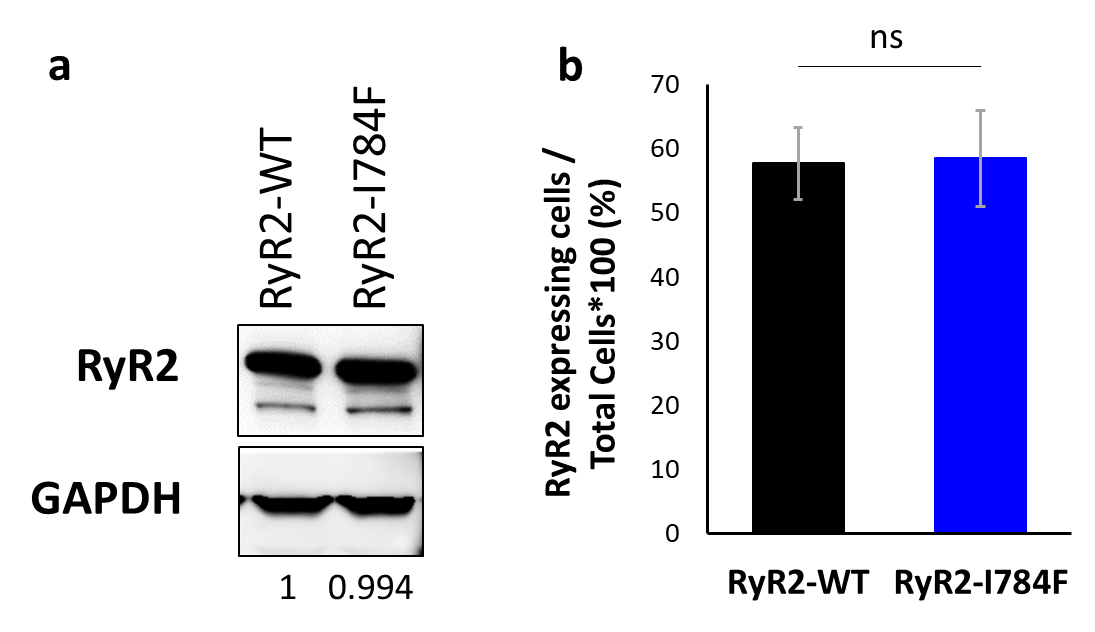
**

**Supplementary Figure S2. Biochemical characterization of recombinant eGFP-WT-hRyR2 or** **eGFP- hRyR2-I784F plasmids. a)** Lysates from HEK293 cells transfected either with eGFP-WT-hRyR2 or eGFP- hRyR2-I784F expressing plasmids were analyzed by immunoblotting using a mouse monoclonal anti-RyR2 antibody (1/1000) and anti-GAPDH antibody (1/2000) to ensure equal loading and transfer (see Supplementary Methods). Membrane was revealed using IR-fluorescence imager. Immunoblot from one representative experiment out of four is shown. **b)** The percentage of caffeine-responding cells was determined as an indicator of the efficacy of transfection. Total number of cells analyzed from five separate experiments in triplicate; ns = not significant.

**Supplementary Figure S3.** **a)** SDS-PAGE of purified RyR2- I784F- SPRY1. **b)** Representative I784F crystals produced by the vapor-diffusion method.

**Supplementary Methods**

**Protein extraction and Western blotting**

HEK293 cells were seeded in 25 cm^2^ dishes and then transfected with 2µg of eGFP-WT-hRyR2 or eGFP- hRyR2-I784F plasmids. Two days after transfection, transfected cells were lysed with a buffer containing 50 mM Tris-HCl (pH 7.5), 150 mM NaCl, 1% Triton X-100, 0.5% sodium deoxycholate, 0.1% SDS and 5 mM EDTA, for one hour at 4°C under gentle stirring, and then centrifuged at 13 200 rpm for 10 min at 4°C. Protein concentration was determined using a PierceTM BCA Protein Assay kit (ThermoScientific, France). Total proteins (20 µg) were separated by 3-8% Tris Acetate polyacrylamide gel electrophoresis and then transferred into a nitrocellulose membrane for 90 min at 35 V. Membranes were saturated with 5% milk-0.1% Tween-Phosphate Buffered Saline (PBS) for 45 min, and then incubated overnight with antibodies against RyR2 (1:2000) (MA3-916, ThermoScientific, France) and α-GAPDH (1:2000) (ab9485, Abcam, Cambridge, United Kingdom). Proteins were detected by enhanced chemiluminescence (Pierce). RyR2, FKBP12.6 and GAPDH expression were quantified with ImageJ.

**Supplementary references**

1. Gaborit, N. *et al.* Regional and tissue specific transcript signatures of ion channel genes in the non-diseased human heart. *The Journal of Physiology* **582**, 675–693 (2007).

2. Haissaguerre, M., Vigmond, E., Stuyvers, B., Hocini, M. & Bernus, O. Ventricular arrhythmias and the His-Purkinje system. *Nat Rev Cardiol* **13**, 155–166 (2016).

3. Robyns, T. *et al.* Clinical and ECG variables to predict the outcome of genetic testing in hypertrophic cardiomyopathy. *Eur J Med Genet* 103754 (2019) doi:10.1016/j.ejmg.2019.103754.

4. Sabater-Molina, M., Pérez-Sánchez, I., Hernández Del Rincón, J. P. & Gimeno, J. R. Genetics of hypertrophic cardiomyopathy: A review of current state. *Clin. Genet.* **93**, 3–14 (2018).

5. McNally Elizabeth M. & Mestroni Luisa. Dilated Cardiomyopathy. *Circulation Research* **121**, 731–748 (2017).

6. Goodyer William R. *et al.* Transcriptomic Profiling of the Developing Cardiac Conduction System at Single-Cell Resolution. *Circulation Research* **125**, 379–397 (2019).

**Full unedited gel for Supplementary Figure S2.**


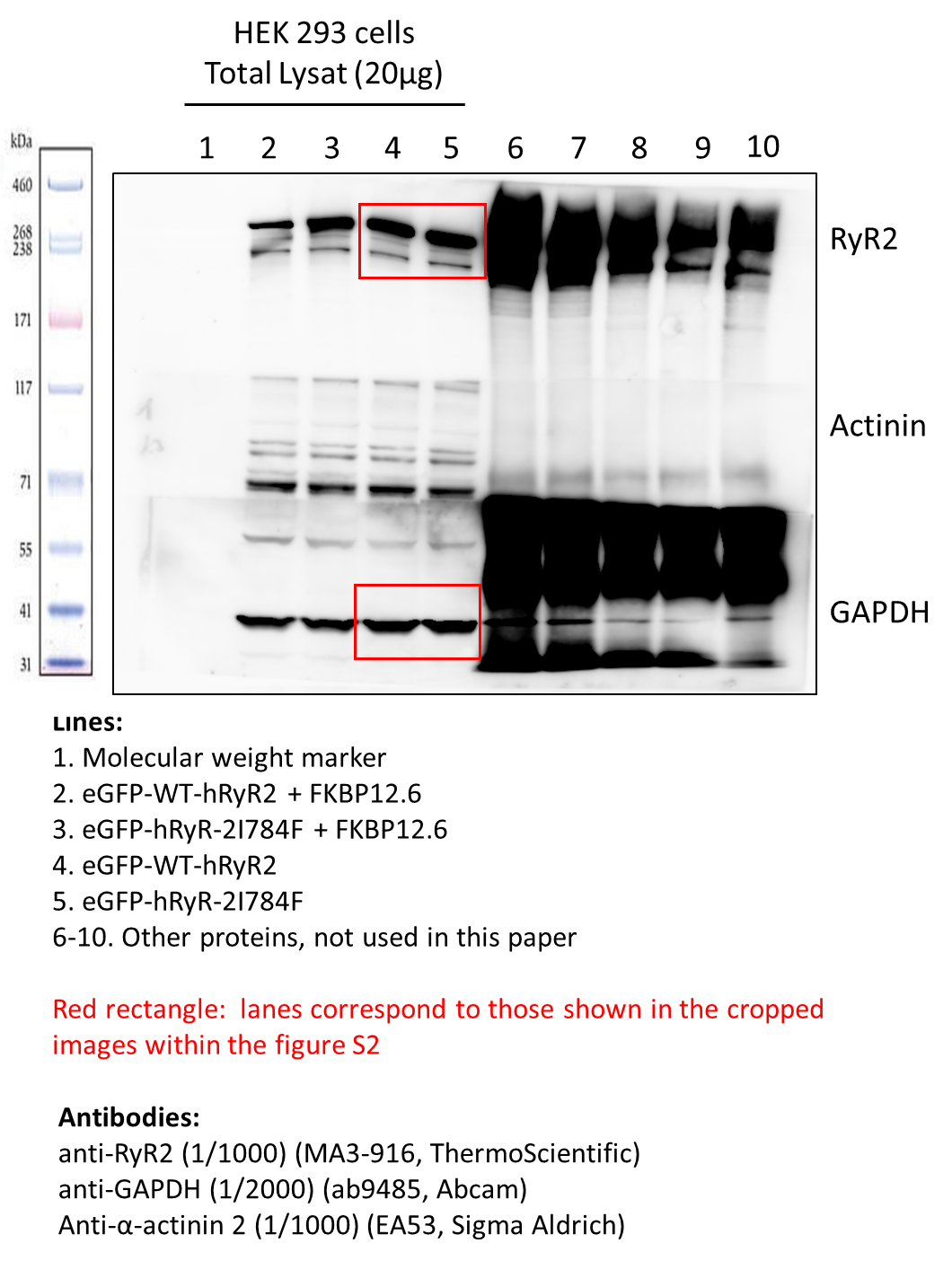


**Full unedited gel for Supplementary Figure S3.**


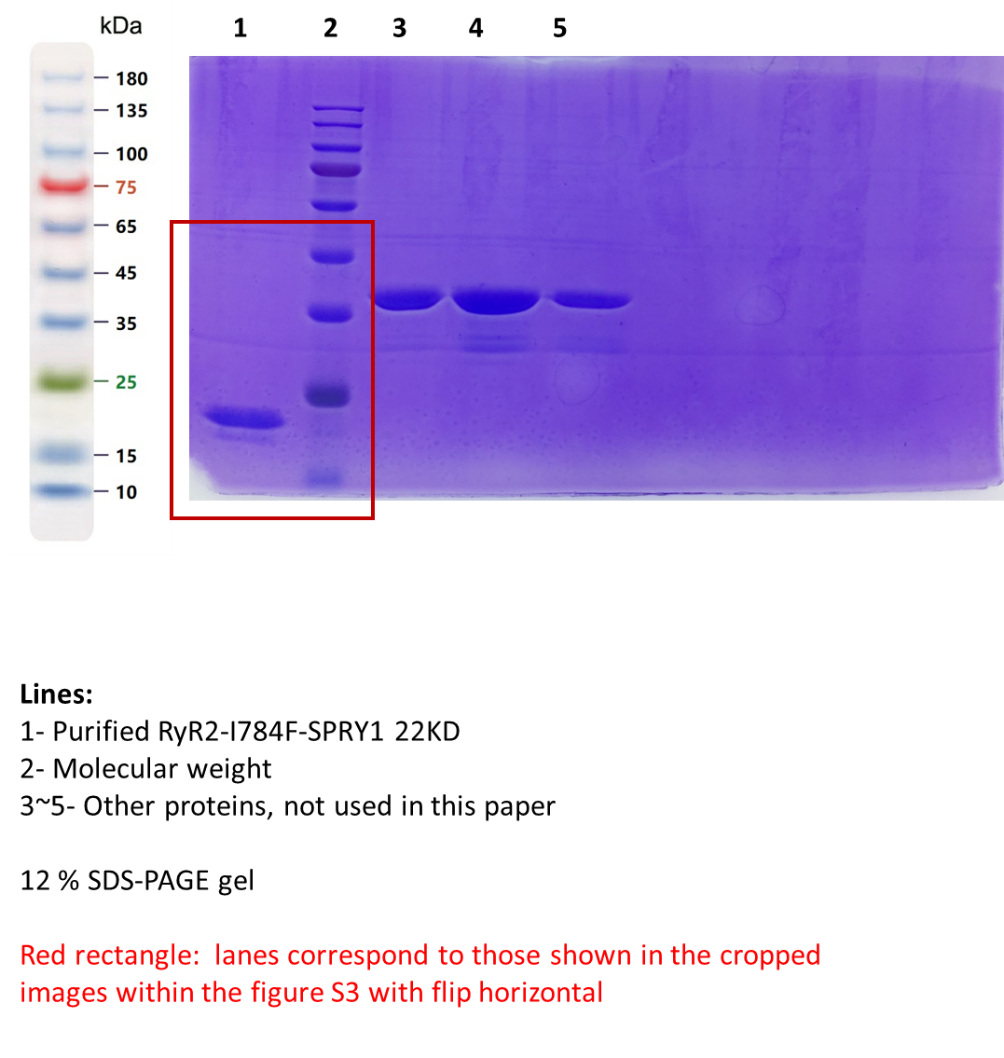

Supplement: Supplementary file 1 — Supplementary Information. [file 41598_2021_84373_MOESM1_ESM.docx]
